# Predictive Values of MRI and PET Derived Quantitative Parameters for Patterns of Failure in Both p16+ and p16– High Risk Head and Neck Cancer

**DOI:** 10.3389/fonc.2019.01118

**Published:** 2019-11-14

**Authors:** Yue Cao, Madhava Aryal, Pin Li, Choonik Lee, Matthew Schipper, Peter G. Hawkins, Christina Chapman, Dawn Owen, Aleksandar F. Dragovic, Paul Swiecicki, Keith Casper, Francis Worden, Theodore S. Lawrence, Avraham Eisbruch, Michelle Mierzwa

**Affiliations:** ^1^Department of Radiation Oncology, University of Michigan, Ann Arbor, MI, United States; ^2^Department of Radiology, University of Michigan, Ann Arbor, MI, United States; ^3^Department of Biomedical Engineering, University of Michigan, Ann Arbor, MI, United States; ^4^Department of Biostatistics, University of Michigan, Ann Arbor, MI, United States; ^5^Department of Radiation Oncology, VA Ann Arbor Healthcare System, Ann Arbor, MI, United States; ^6^Department of Internal Medicine, University of Michigan, Ann Arbor, MI, United States; ^7^Department of Otolaryngology, University of Michigan, Ann Arbor, MI, United States

**Keywords:** MRI, head and neck cancer, radiation therapy, imaging biomarker, adaptive therapy

## Abstract

**Purpose:** FDG-PET adds to clinical factors, such tumor stage and p16 status, in predicting local (LF), regional (RF), and distant failure (DF) in poor prognosis locally advanced head and neck cancer (HNC) treated with chemoradiation. We hypothesized that MRI-based quantitative imaging (QI) metrics could add to clinical predictors of treatment failure more significantly than FDG-PET metrics.

**Materials and methods:** Fifty four patients with poor prognosis HNCs who were enrolled in an IRB approved prospective adaptive chemoradiotherapy trial were analyzed. MRI-derived gross tumor volume (GTV), blood volume (BV), and apparent diffusion coefficient (ADC) pre-treatment and mid-treatment (fraction 10), as well as pre-treatment FDG PET metrics, were analyzed in primary and individual nodal tumors. Cox proportional hazards models for prediction of LRF and DF free survival were used to test the additional value of QI metrics over dominant clinical predictors.

**Results:** The mean ADC pre-RT and its change rate mid-treatment were significantly higher and lower in p16– than p16+ primary tumors, respectively. A Cox model identified that high mean ADC pre-RT had a high hazard for LF and RF in p16– but not p16+ tumors (*p* = 0.015). Most interesting, persisting subvolumes of low BV (TV_bv_) in primary and nodal tumors mid-treatment had high-risk for DF (*p* < 0.05). Also, total nodal GTV mid-treatment, mean/max SUV of FDG in all nodal tumors, and total nodal TLG were predictive for DF (*p* < 0.05). When including clinical stage (T4/N3) and total nodal GTV in the model, all nodal PET parameters had a *p*-value of >0.3, and only TV_bv_ of primary tumors had a *p*-value of 0.06.

**Conclusion:** MRI-defined biomarkers, especially persisting subvolumes of low BV, add predictive value to clinical variables and compare favorably with FDG-PET imaging markers. MRI could be well-integrated into the radiation therapy workflow for treatment planning, response assessment, and adaptive therapy.

## Introduction

Locoregional failure (LRF) remains a clinical challenge for poor prognosis locally advanced squamous cell carcinoma of the head and neck (HNSCC) treated with definitive chemoradiation therapy (CRT) (1). It is important to identify imaging markers of LRF that identify patients and tumor subvolumes that may benefit from intensified locoregional therapy in the form of radiation boost, targeted systemic therapy, or surgical intervention.

We and others have been developing prognostic and predictive imaging markers of PET and MRI for LRF, distant metastases, progression free survival (PFS), and overall survival (OS) (2-20). Retrospective studies of pre-treatment FDG-PET that quantify cellular glucose metabolism have identified metabolic tumor volume (MTV), total lesion glycolysis (TLG), and mean/max standard uptake value (SUV) in MTV as prognostic for LRF, PFS, and OS in HNSCC (2–6). Furthermore, FDG-PET has been incorporated into standard of care work-up and follow-up for HNSCC (7, 8). Functional MRI incorporating diffusion and perfusion parameters is an emerging advanced imaging modality in HNSCC. In particular, apparent diffusion coefficient (ADC) correlates with locoregional and distant progression (9–11). Poorly perfused and low oxygenation tumors have been shown to be associated with LRF and worse survival outcomes (12–18).

Despite this progress, it has been difficult to determine which imaging biomarkers should be used to individualize treatment for the patients with locally advanced HNSCC. Most head and neck cancer imaging studies to date include heterogeneous populations of various disease sites, stages, and prognosis. Few imaging studies investigate how p16 status affects imaging parameters pre- and mid-treatment. With respect to ADC in particular, no study to date has evaluated ADC changes during RT for p16+ vs. p16– tumors. A single study investigated ADC differences between HPV+ and HPV– HNSCC, including only 6 HPV+ patients (8%), and found that pre-treatment ADC in HPV+ HNSCC patients was significantly lower than in HPV– patients (19). Furthermore, at the tumor and subtumor level, there is no report on imaging biomarker differences between tumors with local, regional, or distant failure as site of first failure compared to disease free patients. This is an important issue, as it would help stratify the patients for local or systemic intensified or de-intensified therapy. Finally, poorly perfused tumor subvolumes are largely spatially distinct from areas of high FDG uptake and high restricted water diffusion in the same patients, and the spatial correlation between high glucose metabolism and high restricted water diffusion varies greatly from patient to patient (20, 21). These studies question whether both FDG PET and MRI biomarkers are necessary to guide adaptive RT in HNSCC.

This study aimed to (1) investigate p16+ effects on imaging parameters and their early response rates; (2) assess differences between imaging biomarkers of tumors with local, regional or distant progression and those with no evidence disease (NED), and (3) compare the predictive values of MRI and PET biomarkers. We hypothesized that p16+ status could affect imaging biomarkers and their early response rates, and MRI-based QI metrics could add to clinical predictors of treatment failure more significantly than FDG-PET metrics for local, regional and distant failure.

## Methods

### Patients

Imaging analysis was performed on 54 patients [median age of 61 years; 7 females; 31 p16+ (57%)] with advanced HNSCC who were enrolled in a randomized phase II clinical trial between March 2014 and January 2018 (Table 1). The trial was approved by the Institutional Review Board of the University of Michigan, including a parallel imaging study to investigate the predictive values of QI metrics for tumor progression. Written consent was obtained from all enrolled patients. Eligibility included patients with p16+ T4/N3 squamous cell carcinoma of oropharynx or locally advanced p16– HNSCC if planned to undergo definitive CRT. All patients were evaluated for p16 status by immunohistochemistry. After completion of CRT, patients were followed up every 2–3 months per standard care for oncologic outcomes as well as toxicity. Tumor recurrences were scored as LF, RF, or DF, or a combination thereof.

**Table 1:** Patient characteristics.

**Variable**	**Mean**	**Range**	**Median**
Age	61	31–85	61
Smoking pack years	37	0–150	30
Gross tumor volume			
Primary tumor (CC)	77	10–595	61
Nodal tumor (CC)	22	0.4–242	6
	**Category**	**Count**	**Percentage %**
Primary tumor site	Nasopharynx	3	5
	Oral cavity	6	11
	Oropharynx	35	65
	Larynx	2	4
	Hypopharynx	6	11
	Nasal sinonasal	2	4
p16	Negative	23	43
	Positive	31	57
Smoking status	Never	6	11
	Former	34	63
	Current	14	26
T stage	1	1	2
	2	2	4
	3	6	11
	4	45	83
N stage	0	6	11
	1	3	6
	2	38	70
	3	7	13
Dose	70 in 35 Fx	34	63
	80 in 35 Fx	20	37
Chemo	Carboplatin	26	48
	Cisplatin	28	52

### MRI and PET Acquisition

Patients underwent FDG-PET/CT scans pre-RT within 4 weeks of RT as a part of standard care. Clinical FDG-PET/CT scans were performed on various PET scanners by following the standard clinical protocol (22).

MRI scans were acquired pre-RT (within 2 weeks) and at fraction 10 (20 Gy) as a part of the protocol. All MRI scans were acquired on a 3T scanner (Skyra, Siemens Healthineers), including anatomic, diffusion weighted (DW), and DCE T1-weighted imaging series. All patients were scanned in the treatment position using an individual-patient immobilization 5-point mask and bite block or aquaplast mold as required for treatment. DW images were acquired with spatial resolution of ~1.2 × 1.2 × 4.8 mm and *b*-values of 50 and 800 s/mm^2^ by either a 2D spin-echo single shot echo-planar pulse sequence or a readout segmentation of long variable echo-trains (RESOLVE) pulse sequence that reduced geometric distortion (23). Sixty T1-weighted DCE image volumes were acquired using a 3D gradient echo pulse sequence in a sagittal orientation with voxel size ~1.5 × 1.5 × 2.5 mm during an injection of one standard dose of Gd-DTPA. Post-Gd T1-weighted images were acquired in the axial plane with spatial resolution of 0.875 × 0.875 × 3.3 mm by a 2D fast spin echo sequence with fat saturation.

### Image Analysis and Registration

Blood volume (BV) maps were quantified from DCE-MRI using the modified Tofts model implemented in an in-house imFIAT Analysis Tool, which was validated using a digital reference object (24). ADC maps were calculated from DW images with *b*-values of 50 and 800 to mitigate the perfusion effect by using in-house software that was technically validated in a QIN collaborative project (25). Since using the individual-patient immobilization devices reduced gross movement of head and neck during scanning dramatically, BV and ADC maps were reformatted to match voxel-by-voxel of post-Gd T1-weighted images acquired in the same session using coordinates in DICOM headers. SUV of FDG-PET was calculated. Pre-RT FDG-PET/CT and mid-treatment MR images were co-registered to pre-RT post-Gd T1-weighted images using rigid body transformation and mutual information. Target displacement errors, including image mis-registration and geometric distortion in ADC maps, between image series were assessed and reported previously (20). Reproducibility of BV maps was 16%, which was reported previously (26).

### Tumor Volumes and Subvolumes

Gross tumor volume (GTV) of primary and nodal disease was contoured individually on post-Gd T1-weighted images by treating attending head and neck radiation oncologists and reviewed by the trial PI (MM). For this cohort of patients with locally advanced HNSCC, gross cystic or necrotic regions and tumor invasion into blood vessels occurred in many tumors, and therefore were excluded from the GTVs for following analyses of quantitative image (QI) metrics by applying simple thresholds. For the ADC analysis, a threshold of >2.7 × 10^−3^ mm^2^/s (10% below free water diffusion) was used to exclude gross necrosis and blood vessels, and a threshold of <0.0001 × 10^−3^ mm^2^/s was used to exclude air. Then, a low BV subvolume of the GTV (TV_BV_) was created using a threshold of BV <7.64 ml/100 g reported previously based upon a histogram analysis (16). The low ADC subvolume of the GTV (TV_ADC_) was defined as ADC <1.2 × 10^−3^ mm^2^/s based on an ADC-histogram analysis (20), which is also consistent with the mean ADC reported by others (21). A MTV was defined as FDG SUV >50% of a value averaged over 4 voxels with maximum SUVs (MTV_50_).

### Quantitative Imaging Metrics

QI metrics in tumor volumes and their mid-treatment changes were analyzed for prediction of LF, RF, and DF. Tumor volume metrics included GTV, TV_BV_, TV_ADC_, MTV_50_. Mean values of ADC and BV in GTV excluding blood vessels and necrosis, mean and max SUVs in MTV_50_, and TLG of MTV_50_ were calculated for each primary or nodal tumor as well as for all tumors in each patient.

### Treatment

The patients were randomized to a standard arm of RT (70 Gy in 35 fractions) or an experimental arm. In the experimental arm, a union of the persisting TV_BV_ pre-RT to 2 weeks and persisting TV_ADC_ pre-RT to 2 weeks received 2.5Gy per fraction for the last 15 of 35 fractions. If the union of persisting subvolumes pre-RT to 2 weeks was <1 cc, the patient was entered into an observation arm and treated by the standard RT (70 Gy in 35 fractions). Patients were planned to receive weekly cisplatin 40 mg/m^2^, and patients considered to be cisplatin ineligible were treated with weekly carboplatin AUC2.

### Statistical Analysis

First, we assessed the p16 effect on imaging parameters and parameter change rates at 2 weeks compared to pre-RT using the Mann-Whitney *U*-test. Secondly, we assessed whether MRI and PET biomarkers had similar predictive values for LRF and DF free survival. For the analysis of LRF, most previous analyses considered either LF, RF, or LRF as an event, of which the model was useful for stratification of the patients but not for stratification of the tumors for intensified adaptive RT. Tumor progression could occur in one or a few treated tumors (primary or nodal tumor) or in none. Therefore, we applied Cox proportional hazards models to individual (primary or nodal) tumors for prediction of failure. The individual tumor failure free rate (ITFFR) was defined from the start of RT to the date of progression of the tested (primary or nodal) tumor. ITFFR times were censored for all tumors from a patient at the earlier of DF, death or last follow-up. Whether primary and nodal tumors can be analyzed together was tested for each imaging parameter. To compare the predictive values of MRI and FDG PET biomarkers, imaging metrics were assessed one at a time in models also including p16 as a co-variable, which is the most important clinical variable for LRF (27–29). Distant failure free survival (DFRS) was defined as the time interval from the start of RT to the date of DF. The Cox models were fitted including a single QI metric and clinical stage T4/N3 vs. other (non-T4/N3) as the sole clinical variable (30–32), and entering one imaging parameter at a time. Each QI metric was summed up or averaged over all nodal tumors for volume-related or intensity-related metrics, respectively. In the DFFS model, patients were censored at the first occurrence of any local or regional failure, death or last follow-up. If there were any significant differences of imaging parameters between p16– and p16+ tumors, we considered an interaction term in the Cox model or an analysis in different Cox models as appropriate. Since multiple comparisons were made, *p*-values were corrected using false discovery rate (FDR) control, and corrected *p* < 0.10 were considered significant. Finally, we assessed if there were any significant differences of imaging biomarkers between the tumors that never progressed, those that demonstrated local or regional progression, and those that were locoregionally controlled but metastasized distantly. This landmark analysis used outcomes at 18 months as a cutoff. The tumors were excluded from the analysis if the tumor had local or regional progression after 18 months or the tumor had no progression but the follow-up was shorter than 18 months. As the data were not Gaussian distributed, non-parametric tests were used: Kruskal-Wallis test for the three-group comparison and Wilcoxon rank test for the comparison between local or regional failure and NED. The *p*-values were corrected with FDR control, and <0.1 were considered as significant. Since 37% of the patients received higher doses, we tested the dose effect before performing the proposed analyses.

## Results

### Treatment Failure

This cohort of 54 patients with locally advanced HNSCC had large primary GTVs with a median value of 60.5 cc (range: 10.2–595.2 cc; SD: 86.8 cc; Table 1), which was several times greater than most reported studies (2–6, 9–11, 33). Eleven patients (20%) (3 p16+) have had local recurrence. Nine patients (17%) (2 p16+) have had regional recurrence, including one patient (p16–) who failed regionally at two separate treated lymph node locations, and 2 (1 p16– and 1 p16+) who had RF at the locations of non-enlarged/non-FDG avid nodes before RT. Fourteen patients (7 p16+) had distant failure with or without local and regional failure. All cases with LF or RF alone were confirmed pathologically, and distant metastases were diagnosed pathologically or by overt radiographic presentation. Twelve patients have died of HNC (3 p16+), and one patient died cancer-free of other causes. For the patients who did not have progression at the time of analysis, median follow-up was 24 months (range: 10–58 months).

### Effects of p16 on Imaging Parameters and Change Rates

**Figure 1:**
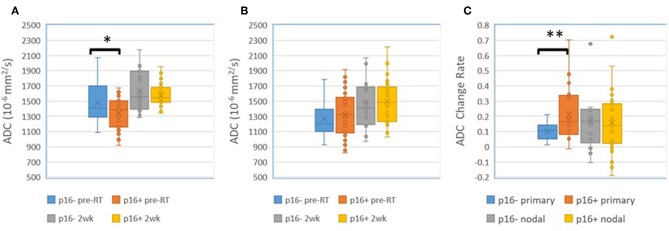
**(A)** Mean ADC in p16– and p16+ primary tumors pre-RT and after 10 fractions (2 weeks) of radiation therapy. **(B)** Mean ADC in p16– and p16+ nodal tumors pre-RT and after 10 fractions (2 weeks) of radiation therapy. **(C)** Mean ADC change rates in p16– and p16+ primary and nodal tumors after 10 fractions of radiation therapy compared to pre-treatment. *p < 0.05, **p < 0.01.

**Figure 2:**
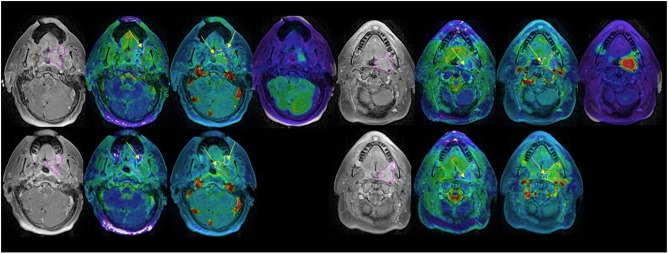
Post Gd T1 weighted images (left), ADC (second left), blood volume (second right), and SUV **(right)** FDG PET pre-RT (top) and after 10 fractions of radiation therapy **(bottom)**. GTV: magenta; low BV subvolume: yellow; low ADC subvolume: cyan; MTV: red. Note persistent low BV and low ADC subvolumes after 10 fractions of radiation therapy (yellow arrows).

We found that both baseline ADC and ADC change after radiation were significantly different between p16+ and p16– primary tumors. The p16– primary tumors had significantly greater mean ADCs pre-RT [1.48 ± 0.05(SEM) μm^2^/ms], and significantly smaller rates of increase after 10 fractions of RT (10.0% ± 1.2%) than p16+ primary tumors (1.34 ± 0.04 μm^2^/ms and 21.2% ± 3.1%, *p* = 0.04, and *p* = 0.009, respectively). However, there was no significant difference in mean ADC between p16– and p16+ nodal tumors pre-RT or at 2 weeks as well as ADC increased rates (*p* > 0.7), see Figure 1. Pre-RT GTVs of p16– primary tumors (75 ± 12.1 cc) as well as change rates at 2 weeks (−16.2% ± 3.9%) were similar to p16+ ones (79.2 ± 18.9 cc, and −16.7% ± 3.3%, respectively). Mean GTVs as well as change rates at 2 weeks for p16– and p16+ nodal tumors were not significantly different (*p* > 0.5), 24.1 ± 8.1 cc and 21.1 ± 5.9 cc of GTVs and −22.4% + 6.7% and −16.5% + 4.5% of change rates for respective p16– and p16+ nodal tumors Also, there was no significant difference in other imaging parameters between p16+ and p16– primary or nodal tumors (*p* > 0.1). Examples of images are shown in Figure 2.

### Predictive Values of MRI and PET Imaging Parameters for Local and Regional Progression

First, we did not detect significant difference in local and regional control rates between two-dose arms yet so that the patients who received different doses were analyzed together. For prediction of local progression, mean ADC pre-RT of primary tumors was the only parameter found significant in a univariate Cox model. Since there was no significant difference in mean ADC between primary and nodal tumors, we combined primary and nodal tumors in a single model (53 primary tumors and 82 nodal tumors). For prediction of ITFFR, considering the p16 effect on mean ADC of primary tumors, the Cox model included p16 status, pre-RT mean ADC, and the interaction of pre-RT mean ADC and p16 status. We found that p16 had a significant effect on tumor control (HR p16+ vs. p16– of 0.21, *p* = 0.005), pre-RT mean ADC had a significant effect in p16– tumors (HR per 1 SD increase in ADC = 1.9, *p* = 0.015) but no effect in p16+ tumors (HR = 1.0, *p* = 1.0). The interaction between p16 status and ADC was not statistically significant (*p* = 0.24, Table 2).

**Table 2:** Cox model of mean ADC effects.

	**Coef**	**HR**	**Lower 95%**	**Upper 95%**	***p*-value**
p16+ effect^*^	−1.578	0.206	0.069	0.618	0.005
ADC effect for p16–	0.645	1.905	1.135	3.198	0.015
ADC effect for p16+	0.000	1.000	0.383	2.611	1.0
ADC^*^p16 effect interaction (ratio of ADC effects between p16+ and p16–)	−0.644	0.525	0.177	1.553	0.2

* *At mean ADC value*.

Since QI metrics other than mean ADC were significantly different between primary and nodal tumors (*p* < 0.05), the QI metrics of nodal tumors were tested separately for prediction of regional failure free rates. In Cox models of 82 nodal tumors with p16 status as a co-variate, GTV pre-RT and at 2 weeks, TV_BV_ at 2 weeks, mean and max SUV in MTV_50_ pre-RT, MTV_50_ pre-RT, TLG pre-RT, and change in GTV at 2 weeks vs. pre-RT were significant with *p* < 0.07 with FDR control, see Table 3. It is interesting to note that GTV pre-RT and at 2 weeks as well as mean SUV and TLG pre-RT have the highest c-index (> 0.9). However, MTV_50_ and TLG as well as TV_BV_ were strongly correlated with GTV pre-RT (range of *r* between 0.88 and 0.90), suggesting that these metrics are not independent of GTV. The mean and max SUV in MTV_50_ were strongly correlated each other (*r* = 0.98) but modestly correlated with GTV pre-RT (range of *r* between 0.65 and 0.67).

**Table 3:** Cox models for RFFS.

	**Hazard ratio**	**C index**	**Pr(>|z|)nominal *P*-value**	***P*-value with FDR adjustment**
GTV preRT	1.120	0.90	0.02	0.05^*^
GTV 2 weeks	1.142	0.91	0.001	0.01^*^
TV_BV_ 2 weeks	1.514	0.88	0.005	0.02^*^
MTV_50_ preRT	1.524	0.88	0.03	0.07^*^
Mean SUV preRT	1.406	0.90	0.005	0.02^*^
Max SUV preRT	1.238	0.88	0.008	0.02^*^
TLG preRT	1.073	0.90	0.001	0.01^*^
Change in GTV	1.288	0.72	0.004	0.02^*^

*82 nodal tumors were included in the analysis. Change in GTV was after 10 fractions of RT compared to preRT*.

* *Indicates significant*.

### Predictive Values of Imaging Biomarkers for Distant Progression

For prediction of distant progression, Cox models identified that TV_BV_ of primary tumors at 2 weeks, total TV_BV_ of all nodal tumors pre-RT and at 2 weeks, total GTV of all nodal tumors at 2 weeks, mean and max SUV of all nodal MTV50 pre-RT, and TLG pre-RT of all nodal tumors had a nominal *p* < 0.05 without FDR. With FDR control, total GTV of all nodal tumors at 2 weeks, mean and max SUV of all nodal MTV50 pre-RT, and TLG pre-RT had a *p* < 0.1, see Table 4. We tested whether the significant predictors could provide any complimentary information to clinical stage of T4/N3 and the sum of all nodal GTVs at 2 weeks for prediction of DF, and found that neither total TV_BV_, nor mean and max SUV, nor total TLG of all nodal tumors had a *p* < 0.3, and only TV_BV_ of primary tumors at 2 weeks showed marginally significant (*p* = 0.06).

**Table 4:** Cox models for DFFS.

	**Hazard ratio**	**Pr(>|z|)**	***P* with FDR**
**PRIMARY TUMOR**			
Mean BV 2 weeks	0.797	0.008	0.18
**NODAL TUMOR**			
Sum of TV_BV_ preRT	1.290	0.05	0.15
Sum of GTVs 2 weeks	1.091	0.01	0.07*
Sum of TV_BV_ 2 weeks	1.290	0.02	0.12
Mean SUV of all MTV_50_ pre	1.363	0.01	0.09*
Max SUV of all MTV_50_ pre	1.233	0.01	0.09*
TLG of all MTV_50_ pre	1.054	0.02	0.09*

T4/N3 was included as a co-variable. *Indicates significant.

### Imaging Biomarkers for Differentiation of Tumors With LF (or RF), DF, and NED

**Figure 3:**
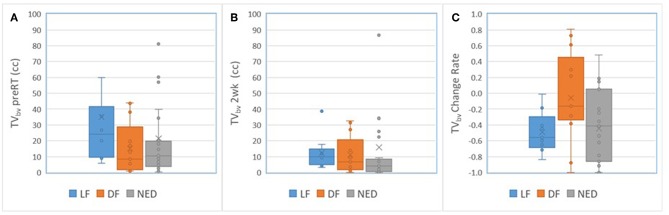
Box and Whisker plots of the subvolumes of low BV in primary tumors with local failure (blue), distant failure (orange), and no evidence disease (gray) pre-RT **(A)** and after 10 fractions of radiation therapy **(B)**. **(C)** Plots the change rates of the low BV subvolumes after 10 fractions of radiation compressed to pre-RT.

For primary tumors, the subvolumes of low BV pre-RT showed a descending trend from LF, DF, and NED with a marginally significant *p*-value of < 0.06 without FDR control, see Table 5. Figure 3 shows the subvolumes of low BV of primary tumors with LF, DF, and NED pre-RT and at 2 weeks as well as its change rates after 10 factions of RT. Post *ad hoc* analysis showed that the change rates of low BV subvolume were significant smaller in primary tumors with DF (−0.05% ± 0.16%) than tumors with LF (−0.49 ± 0.08%) and tumors with NED (−0.45 ± 0.09%) with *p* values of < 0.03 and < 0.015, respectively.

**Table 5:** Differences of imaging biomarkers among tumors with LF (or RF), DF, and NED.

	**Median** **DF** **(*n* = 12)**	**Median** **LF** **(*n* = 10)**	**Median** **NED** **(*n* = 30)**	**KW test** ***P* value**	**KW test** ***P* value with FDR**	**WR test** ***P* value**	**WR test** ***P* value with FDR**
**Primary tumor**							
TV_BV_ pre	11.37	24.24	7.10	0.06	0.3	0.08	0.3
	**DF** **(*****n*** **= 17)**	**LF** **(*****n*** **= 8)**	**NED** **(*****n*** **= 57)**	**KW test** ***P*** **value**	**KW test** ***P*** **value with FDR**	**WR test** ***P*** **value**	**WR test** ***P*** **value with FDR**
**Nodal tumor**							
GTV pre	8.68	32.14	5.78	0.01^*^	0.1^*^	0.07	0.5
GTV 2 weeks	6.57	24.94	3.97	0.02^*^	0.1^*^	0.08	0.5
Change in GTV	−1.07	−1.27	−0.44	0.83	0.9	0.8	0.8
TV_BV_ pre	2.77	4.77	1.77	0.02^*^	0.1^*^	0.2	0.5
TV_BV_ 2 weeks	1.94	7.40	1.18	0.06	0.1	0.2	0.5
Mean ADC pre	1.38	1.40	1.19	0.06	0.1	0.9	0.9
Mean ADC 2 weeks	1.55	1.64	1.35	0.02^*^	0.1^*^	0.4	0.6
Mean BV pre	8.26	10.11	10.21	0.46	0.6	0.5	0.6
Mean BV 2 weeks	8.83	13.33	10.93	0.04^*^	0.1	0.04^*^	0.5
Mean SUV of MTV_50_ pre	3.91	5.38	2.35	0.02^*^	0.1^*^	0.3	0.5
max SUV of MTV_50_ pre	5.96	7.92	3.50	0.03^*^	0.1	0.3	0.6
TLG of MTV_50_ pre	0.718	3.245	0.420	0.11	0.2	0.3	0.5

*Tumor volume is a unit of cc. ADC is in unit of 10^−3^ mm^2^/s. BV is in unit of (ml/100 g). SUV of FDG is in unit of g/ml. TLG is in unit of 100 g*.

* *Indicates significant*.

**Figure 4:**
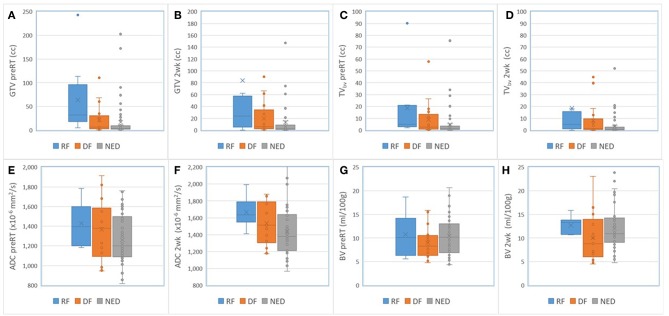
Box and Whisker plots of the GTVs **(A,B)**, the subvolumes of low BV **(C,D)**, mean ADC **(E,F)**, and mean BV **(G,H)** of nodal tumors with RF (blue), DF (orange), and NED (gray) pre-RT and at 2 weeks.

**Figure 5:**
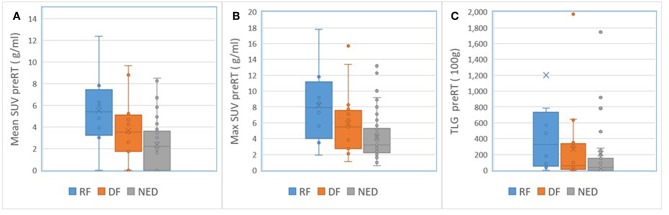
Box and Whisker plots of the mean SUV **(A)**, max SUV **(B)**, and TLG **(C)** of nodal tumors with RF (blue), DF (orange), and NED (gray) pre-RT.

For nodal tumors, GTV pre-RT and at 2 weeks, the subvolume of low BV pre-RT, mean ADC at 2 weeks, mean BV at 2 weeks, and mean/max SUV of MTV_50_ pre-RT were different among DF, RF, and NED groups with *p* < 0.05 without FDR control and *p* ≤ 0.1 with FDR control, see Table 5. Again, GTV of nodal tumors was a strongest parameter to differentiate the three groups with different outcomes. Regarding the difference between DF and RF groups, only mean BV values at 2 weeks had a *p* < 0.05 without FDR control but *p* > 0.1 with FDR control. Figure 4 shows GTVs, the subvolumes of low BV, mean ADC, and mean BV of nodal tumors with RF, DF, and NED pre-RT and at 2 weeks. Figure 5 shows mean SUV, max SUV, and TLG of nodal tumors with RF, DF, and NED pre-RT.

## Discussion

In this study, we investigated p16 effects on MRI and PET QI metrics, imaging biomarker differences as a function of tumor control (local, regional, or distant), and the predictive values between MRI and PET biomarkers for tumor progression in locally advanced poor prognosis HN cancers. Our cohort of patients had large tumor volumes compared to previously reported literature (2–6, 9–11, 33). We found the p16– primary tumors had elevated ADC values pre-RT and low early response rates compared to p16+ tumors; the latter of which has not been previously reported. Also, high mean ADC value pre-RT is a hazard for local and regional failure of p16– tumors. Multiple MRI and PET imaging parameters (including GTV, ADC, BV, SUV, and TLG) predicted RF and DF, but the nodal GTV defined on anatomic MRI was the strongest predictor. Most interesting, we report for the first time that the persistent low BV in primary and nodal tumors during the early course of CRT is associated with high-risk for distant failure. In order to identify patients who may benefit from intensified local therapy in the form of a radiation boost or surgical intervention, or from intensified systemic therapy (30, 34), we analyzed the significant imaging predictors found in Cox modeling for differentiation of the tumors that were controlled compared to those with LF, RF, or DF. The performance of MRI related parameters is stronger than PET parameters. Although PET is a part of standard care, MRI could play an important role from treatment planning, to early response assessment, and boost target definition.

We found a p16 effect on ADC and ADC change rates during the early course of RT. The p16– primary tumors had significantly greater mean ADC values pre-RT and smaller increases in ADC after 2 weeks of CRT than p16+ primary tumors. Furthermore, the p16– tumors from patients with local or regional failure had significantly greater mean ADC values pre-RT and mid-treatment than those from disease free patients. These results are consistent with previous reports that the pre-RT high ADC is negatively prognostic for HN cancers (9–11). A recent study shows that ADC is significantly and inversely correlated with cell density but also significantly and positively correlated with the percentage area of stroma in laryngeal and hypopharyngeal carcinoma (35). The former finding has been reported previously in animal studies, prostate cancer and lymphomas (36–39), and is related to restricted water diffusion due to high cellularity. The latter finding suggests that a large percentage area of stroma in HN cancers is associated with a high ADC. Stroma has been shown to be negatively prognostic in several cancers, to promote tumor growth and invasion, and to potentially protect tumors from delivery of chemotherapy (40–45). ADC behaviors in the p16– tumors could be explained by their increased stroma. HPV-related oropharynx cancers are histologically basaloid in histology with significant tumor lymphocytic infiltration, which is associated with improved prognosis (46, 47) and decreased ADC. ADC, although a promising QI metric for differentiation of local and regional failure, and even distant failure, is affected by multiple biologic and physiologic factors, including cell density and stroma as well as cyst and necrosis (In this study, we excluded grossly cystic and necrotic regions for QI metric analysis).

The low BV in primary tumors and persisting during the early course of RT have reported previously to be associated with LF (12–17). However, there is no report that the low BV and its low response rate in HNSCC during the early course of RT is associated with DF. The subvolumes of low BV in primary tumors show a descending trend from LF, to DF and NED. The response rate of low BV could be used to differentiate the tumor at high-risk for LF or DF from NED, and thereby adapting intensified local or systematic therapy for the patients with different progression risks.

Pretreatment FDG QI metrics, including MTV, TLG and mean/max, have been reported to be correlated with PFS and OS in the patients with HN cancers treated with CRT (3–6). We found that the high mean/max SUV and large TLG in nodal tumors were risk factors for nodal failure, and that the sum of TLG over all nodal MTVs was a negative prognostic factor for DFFS, which is consistent with several previous reports (2–4). Although TLG accounts for both the size and SUV of MTV, we found that nodal TLG was strongly correlated with MRI-defined GTV, and nodal GTV was the strongest predictor for RF in our study. For prediction of RF and DF, several other MRI parameters (including GTV, ADC, and BV) perform as well as FDG PET related parameters. When including T4/N3 and total nodal GTV in the Cox model, no other imaging parameters including PET were found to be significant. Finally, there were no FDG PET related parameters that could predict LF.

Radiomics analysis of CT and PET features is another area of imaging analysis that could provide complimentary information to the present study. Radiomics analysis that extracts the large amounts of quantitative textural features from CT, PET, and MRI has been investigated for the prediction of local control, PFS, and OS in head and neck cancers (48–52). Through the feature selection and reduction processes, a small number of features have been found to have prognostic or predictive value. These features include general categories of statistical energy, shape compactness, gray level non-homogeneity, and gray level non-uniformity. These features may represent different tumor phenotypes. However, it is hard to link the feature to tumor physiology, pathology and biology. Furthermore, radiomics approaches require a large amount of high quality image data, and high-throughput.

A limitation of the present analysis includes RT boost of tumor subvolumes with persistent low BV and low ADC on our clinical trial. This could affect QI metrics that are identified for prediction of treatment failure. We will perform this analysis on patients who are on the standard treatment arm when the trial is completed and the data have matured. Nevertheless, we found that persistent low BV in primary and nodal tumors carries a high-risk for nodal and distant failure, the low response rate of low BV has a high-risk for distant failure, and the low response rate of ADC is for p16– primary tumors. MRI derived biomarkers perform at least as well as FDG PET defined ones. As MRI based planning is already well-integrated into radiation therapy, our findings suggest that MRI based response assessment will be a valuable guide in adaptive radiation therapy.

## Data Availability Statement

The image data that were collected in the patients with at least 10 months follow-up in the study are included in the manuscript/supplementary files.

## Ethics Statement

The studies involving human participants were reviewed and approved by the institute review board of University Michigan. The patients/participants provided their written informed consent to participate in this study.

## Author Contributions

All authors contributed significantly for study design, patient enrollment, image acquisition and analysis, statistic analysis, data interpretation, or writing.

### Conflict of Interest

YC is co-owner of a US patent of No. 61/656,323, which is entitled “The subvolume identification for prediction of treatment outcome”. The remaining authors declare that the research was conducted in the absence of any commercial or financial relationships that could be construed as a potential conflict of interest.
